# Neonatal hearing screening: modelling cost and effectiveness of hospital- and community-based screening

**DOI:** 10.1186/1472-6963-6-14

**Published:** 2006-02-23

**Authors:** Eva Grill, Kai Uus, Franz Hessel, Linda Davies, Rod S Taylor, Juergen Wasem, John Bamford

**Affiliations:** 1 Department of Physical Medicine and Rehabilitation, University of Munich, Germany; 2 Human Communication and Deafness Group, University of Manchester, UK; 3 Institute for Health Care Management, University of Duisburg-Essen, Germany; 4 School of Psychiatry and Behavioural Sciences, University of Manchester, UK; 5 Department of Public Health and Epidemiology, University of Birmingham, UK

## Abstract

**Background:**

Children with congenital hearing impairment benefit from early detection and management of their hearing loss. These and related considerations led to the recommendation of universal newborn hearing screening. In 2001 the first phase of a national Newborn Hearing Screening Programme (NHSP) was implemented in England. Objective of this study was to assess costs and effectiveness for hospital and community-based newborn hearing screening systems in England based on data from this first phase with regard to the effects of alterations to parameter values.

**Methods:**

Design: Clinical effectiveness analysis using a Markov Model. Outcome measure: quality weighted detected child months (QCM).

**Results:**

Both hospital and community programmes yielded 794 QCM at the age of 6 months with total costs of £3,690,000 per 100,000 screened children in hospital and £3,340,000 in community. Simulated costs would be lower in hospital in 48% of the trials. Any statistically significant difference between hospital and community in prevalence, test sensitivity, test specificity and costs would result in significant differences in cost-effectiveness between hospital and community.

**Conclusion:**

This modelling exercise informs decision makers by a quantitative projection of available data and the explicit and transparent statements about assumptions and the degree of uncertainty. Further evaluation of the cost-effectiveness should focus on the potential differences in test parameters and prevalence in these two settings.

## Background

Between one and two children per 1000 live births have a moderate or greater bilateral permanent hearing loss [1-3]. Children with congenital hearing impairment benefit from early detection and management of their hearing loss [4,5]. The neurological development of auditory pathways requires acoustic stimulation in the first 18 months of life [6,7]. Communication deficits due to hearing impairment not discovered within the first two years are not easily recovered by later rehabilitation. The consequence may be delayed development of speech and language as well as of other cognitive and social functions. This delay is already measurable in the first 3 years of life [8].

These and related considerations led to a comprehensive review of the possible role of newborn hearing screening in the UK [9]. The review recommended the introduction of newborn hearing screening and in 2001 the first phase of a national Newborn Hearing Screening Programme (NHSP) was implemented in England; all areas of the country are expected to be covered by 2005/6.

The first implementation phase of the Newborn Hearing Screening Programme (NHSP) includes sites where the screening is performed by Health Visitors at a home visit, usually at 10 days of age. This model is called 'community-based screening,' in contrast to the 'hospital based' model where babies are screened in maternity hospital by a new cadre of screeners prior to discharge with follow-up of missed cases in a variety of ways.

The evaluation of the first phase of the NHSP, commissioned by the Department of Health in accordance with National Screening Committee (NSC) policy, included a comparison of hospital-based and community-based screening as a main comparison of interest. The measures on which differences between the two models might *a priori *be expected are screen performance, maternal satisfaction or anxiety, and costs. The NSC and others have been clear that a national screen for newborn hearing is desirable, with national quality assurance processes and ongoing audit. The policy question is the extent to which a national screen could encompass the two different models of delivery, and if it could, on what basis areas might be permitted or encouraged to select one or the other model. As the first step, the NHSP Steering Group wished to verify that the data on screen performance, maternal satisfaction/anxiety, and costs did not argue strongly against the community-based model.

The aim of the modelling presented here was to determine the costs and effectiveness for hospital and community-based systems of Neonatal hearing screening in England and Wales with a special regard to the effects of reasonable alterations to parameter values.

## Methods

We modelled the cost-effectiveness of the two screening systems, hospital- and community-based screening using some already-available costs data and screen performance data from the first phase implementation, data from the published literature on newborn hearing screening, and further data collection on costs from the first phase of the NHSP. We used a modified version of a decision-analytic model which has been developed for a German Health Technology Assessment funded by the German Federal Ministry of Health [10].

We estimated absolute and incremental costs and effectiveness of two newborn hearing screening settings. The recommendations of the Panel on Cost-Effectiveness in Health and Medicine were followed [11]. Target population is all newborn infants. Health effects are presented in form of number of quality weighed detected child months (QCM), and true positive and false positive diagnoses at certain developmentally important ages (6 and 12 months). If a hearing impairment was diagnosed within the first month after birth, the baby added six QCM at the age of six months. If the child's hearing loss was diagnosed (strictly, identified) at the age of five months, s/he added only one detected child month at age six months. QCM, true positives and false positives were reported at the age of 6 and 12 months and with a time horizon of 120 months. Child months which were added until the age of 6 months were multiplied with a weight of 1, child months added after the age of 6 months were multiplied with decreasing weighting. This was to ensure the interval property of the outcome despite the fact that the incremental benefit of detecting a hearing problem decreases with time.

### The model

**Figure 1:**
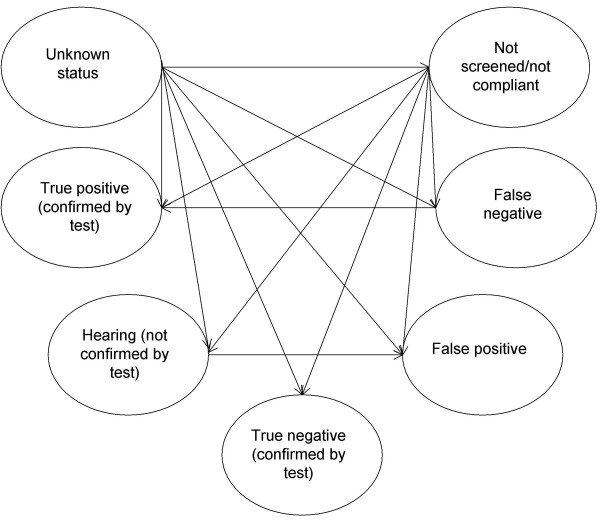
Model figure.

A state-transition (Markov) model [12] was developed to characterise the process of screening and diagnosis through all possible stages (*see *Figure 1). A child can be in one of the following states:

- Unknown status

- Healthy (hearing) confirmed by diagnostic test or screening – true negative

- Healthy (hearing) not confirmed by diagnostic test

- Hearing impaired confirmed by diagnostic test or screening – true positive

- Thought to be healthy (hearing) but hearing impaired – false negative

- Thought to be hearing impaired but healthy (hearing) – false positive

- Not followed up/not compliant

The model starts with a cohort of newborns being of unknown status and applies transition probabilities recursively to simulate how children progress through different states. In each cycle (lasting one month) children can undergo several possible transitions which accrue costs and utility weights. Ultimately all children from the initial cohort are diagnosed as healthy or as impaired or, if they are healthy, some remain 'undiagnosed' (but with true state healthy).

#### Data and assumptions

A predefined and externally reviewed literature search on newborn hearing screening on all relevant electronic databases has been performed. Search strategy and methods have been reported in detail elsewhere [13]. Detected publications were scored according to a standardised questionnaire and included or not. All assumptions made and parameters used are shown in Table 1.

Prevalence of congenital hearing disorders was derived from comprehensive literature searches. The probability of hearing children presenting with falsely suspected hearing disorder was estimated by a panel of experts. The probability of being detected at a certain age without screening was estimated from a survey of activity in an area of Germany in 1998 and 1999 [14]. Positive predictive values were calculated from the empirical yield data. In order to account for the heterogeneity of study sites, positive predictive values were pooled using a random effects model [15]. Test parameters have then been calculated using Bayes' theorem.

The slope of the weighting function was estimated by experts making the following assumptions: each month detected before the age of 6 months is weighted with 1, on the general assumption that children detected (and treated) within the first 6 months of life can develop typical speech and language abilities. If not detected within the first 12 months, profoundly and severely impaired children will end up with a weight of 0.85, and moderately impaired children with a weight of 0.90. Presuming that 50% of the children with permanent congenital hearing disorders are moderately impaired gives a weight of 0.875 for every month which is detected after the first birthday. The weights between 6 and 12 months were calculated by linear extrapolation.

### Model assumptions

Screening and diagnostic procedures are presented under the assumption of conditional independence, i.e. test parameters are independent of the prevalence of the condition and test results of diagnostic testing are independent of test results of screening procedures. This is plausible because screening and diagnostic testing are based on different testing principles.

### Screen performance data and costs

Screen performance data and costs for screening and diagnosis have been derived from empirical data from the NHSP first wave sites.

All community-based areas – East Sussex, Shropshire, Wiltshire (Bath) and Wiltshire (Swindon) – and all hospital-based areas that had started NHSP before 1^st ^May 2002 – Avon, Barnsley, Bradford, Buckinghamshire, Dewsbury, Manchester, North Staffordshire, Northumberland, and Oxford – were included in the study. Four community-based areas and seven hospital-based areas were able to provide data. Table 2 gives the annual birth rates of the included areas.

The following data have been provided by the 11 sites and included into the cost/effectiveness calculation: Screen performance data (number of screened, number of referrals, number of true cases), staff grade and full-time equivalent (screeners, local coordinator, team leader, clerical staff), quantity, make and model of screening equipment, quantity, make and model of computers and printers, quantity and make of consumables, travel costs (exclusive travel associated with training) and any additional costs (e.g. recruitment, refurbishing rooms, stationary). Additional information was obtained from National Health Service salary scales, the National Health Service Rehabilitation Services Catalogue (screening equipment and consumables), the Medical Research Council Institute of Hearing Research for calibration costs, IT costs and training costs. Additionally, training costs during screening and IT training have been obtained. Audiology services reported costs for 10 consecutive referrals and all true cases for audiology follow-up costs.

#### Screen performance data

Incidence and prevalence of congenital permanent bilateral hearing loss were assumed equal.

#### Staff costs

To calculate salaries, midpoint was taken.

Within the community model, health visitors' time for NHSP was estimated at 1%. This estimate was based on a Health Visitor screening on average 1.3 children per week and spending ca 20 minutes on the screen, which is based on data from the sites and Netten et al [16].

National insurance and superannuation was taken as 13%.

Non-staff related costs refer to the overheads, building capital and equipment costs associated with running audiology services. Most NHSP services use a number of different facilities to deliver the different components of the programme and do not have these figures readily available. Hence, to determine these costs, the following steps have been taken: Allowances for indirect overheads (the costs of the support services such as human resources, finance and estates required to carry out the services main functions) have been taken as fixed cost of £2216, and building capital (the costs assigned to treatment and non-treatment space) relative to the level of pay scale based on Netten et al [16].

Direct overheads i.e. the costs associated with lighting, heating and cleaning were assumed to be 11% of the sum of staff costs, indirect overheads and building capital. This was based on previous studies carried out in hospital settings where the direct overheads were found to account for 4% to 18% (midpoint 11%) of total costs [17,18]. As there are no data available, this was equally assumed for community settings. Costs associated with staff turnover have not been included. Staff costs for 10 years were calculated based on the first year costs, except for the Team Leader's post which is included for the first 2 years only and has not been included for the following 8 years.

#### Equipment and IT costs

When equipment is totalled over 10 years, a 5% annuity for each year of life has been allowed for. VAT at 17.5% has been included.

#### Consumable costs

The sites provided information of the quantity of consumables they used in November 2002 and prices obtained from NHS Purchasing and Supply Agency. The figure was multiplied by 12 for the whole year cost. VAT at 17.5% has been included.

#### Calibration costs

Calibration costs were based on the manufacturers' specifications.

#### Travel costs

Only staff travel costs directly associated with the screen were included. Data was obtained from the proformas filled in by the Team Leaders.

#### Training costs

*Initial training cost *calculation was based on cost of attending, cost of conducting the training, venue costs. Cost of attending and conducting the training consist of travel and accommodation costs and cost of time spent by participants and deliverers. Cost of time spent was calculated as number of days attending/delivering training divided by number of workdays per year multiplied by annual salary. Data was obtained from the proformas filled in after each training event. *Refresher training cost *calculation was based on an assumption that refresher training would be 0.5 day a year per screener.

#### Costs of audiological follow-up of screen referrals (false positives)

We assumed the typical audiological assessment which confirms false positive status consisted of an ABR (Automated Evoked Brainstem Response). We assumed a cost of £160 per referral.

#### Societal costs

Costs to the families associated with NHSP screening (travel costs, time off from work, childminding costs) were not included.

Costs and their standard deviations were calculated separately for both settings and weighted with the number of children screened per site.

#### Discounting

Future costs were discounted at a rate of 6% per year, future effects at a rate of 1.5% per year. Yearly discount rates have been converted to monthly discount rates.

#### Sensitivity analysis

One-way and multiple sensitivity analyses were performed on all relevant parameters.

Multivariate simulations were used for probabilistic modelling (Monte Carlo). The simulation associates with each of the model variables a probability density function which represents our uncertainty about a fixed but unknown value. The ranges for test parameter estimates derived empirically and from the literature assumed beta distribution based on available ranges of estimates, and ranges for empirical cost data assumed gamma distribution. The model was evaluated for 1,000 trials.

As the number of sites was small the parameter estimates were estimated in a context of uncertainty. We wanted to evaluate the impact of extreme parameter changes on outcome and decision between alternative settings. As described by Felli and Hazen [19], Monte Carlo simulation was performed on one parameter at a time allowing for the input of extreme values, keeping the other parameters fixed at their baseline level. This analysis was done for prevalence, sensitivity, specificity, coverage and costs. The aim was to show if there is any variation in the input parameter that might result in a change of preference between sites in comparison to the baseline result. One setting can be defined as more cost effective than another if it is (a) less costly and at least as effective, (b) more effective and no more costly, (c) more costly and more effective and its additional costs per unit of effectiveness are considered worth paying, (d) less costly and less effective and the additional costs per extra unit of effectiveness for the alternative setting are not considered worth paying. One unit of effectiveness is defined as one quality weighted detected child month (QCM). The specific goals of the extremes analysis are

- to show the probability that one setting (eg. hospital) is more cost-effective than the other under the assumption that the two sites differ in one parameter, and

- to indicate which difference in a certain parameter between sites might result in substantial differences in costs.

This was achieved by the following procedure: The simulation was run twice with all parameters except one held fixed, the first time with the extreme high estimate of the parameter, the second time with the extreme low estimate of the parameter. This resulted in "high" and "low" estimates for costs and QCM for each setting. Differences of costs and QCM were then calculated using the "high" estimate for hospital and the "low" estimate for community and vice versa. This was done for each of the parameters mentioned. If QCM between hospital and community did not vary, only cost differences were calculated. If both costs and QCM varied the resulting distributions in mean differences of costs and QCM were combined using the Net Benefit Approach [20]. The Incremental Cost Effectiveness Ratio (ICER) is defined as the additional average cost of producing one more unit of effectiveness, here the additional cost for one more QCM achieved in one of the settings, eg. in hospital. Health care planners might decide on a ceiling value λ for these additional costs so that one setting should replace another setting only if the ICER is below this λ. From the distributions of cost and effectiveness differences the probability that one setting is cost-effective compared to another is calculated depending on a range of values for the ceiling ratio λ and presented in the form of a cost-effectiveness acceptability curve [21,22]. The probabilities presented in this curve can be used for formal statistical inference.

Data 3.5 (TreeAge Inc.) was used to construct and run the Markov model and Excel (Microsoft Corp.) was used to validate the model and to perform the Monte Carlo simulations.

## Results

**Figure 2:**
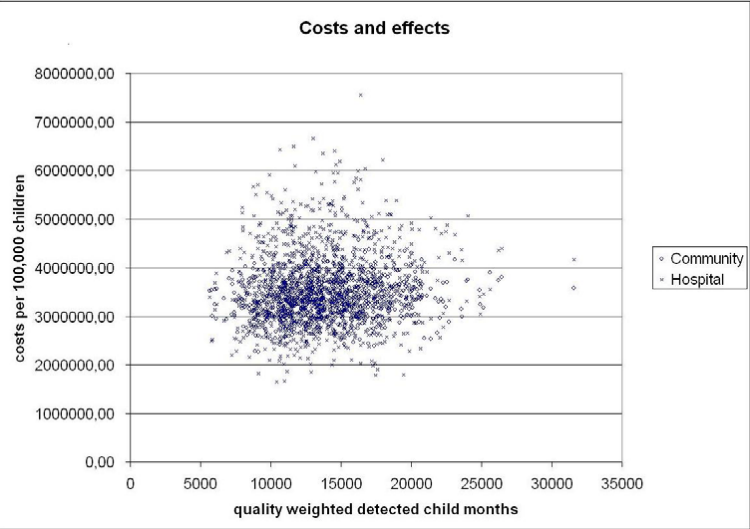
**Costs and effectiveness of screening in hospital and community sites**. Results of probabilistic Monte Carlo simulation (1000 trials).

We modelled costs and effectiveness of universal newborn hearing screening in two different settings. As test parameters were held to be constant across hospital and community sites there was no difference in effectiveness, only in costs. Both hospital and community settings yielded 134 true positive cases (89% of all cases) and 794 quality weighed detected child months (QCM) at the age of 6 months with total costs of £3,690,000 per 100,000 screened children in hospital and £3,340,000 in community. Table 2 shows the annual birth rates of the participating areas. Table 3 and Table 4 show the results of base case and one way sensitivity analysis. Costs per QCM were higher by £25 in hospital-based sites. Sensitivity analysis showed that prevalence had the most important influence on costs per weighted detected child month. Lower prevalence would result in substantial higher costs for each site and in higher incremental costs. The model was, however, rather insensitive to large variations of the other test parameters. Since incremental effectiveness was set zero for base case and sensitivity analysis, the ICER (incremental cost effectiveness ratio) was not available. Figure 2 shows the results of the Monte Carlo simulation. Costs would be lower in hospital sites in 48% of the trials.

### Results of extremes analysis

**Figure 3:**
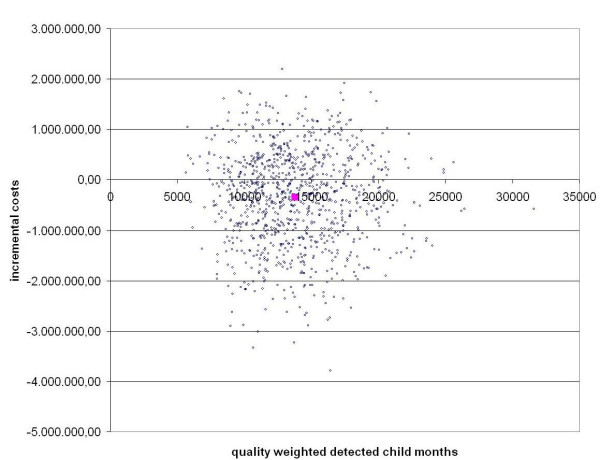
**Incremental costs between hospital and community sites, Monte Carlo simulation with 1000 trials**. Negative incremental costs indicate higher costs in community sites. The solid dot shows the base case result.

**Figure 4:**
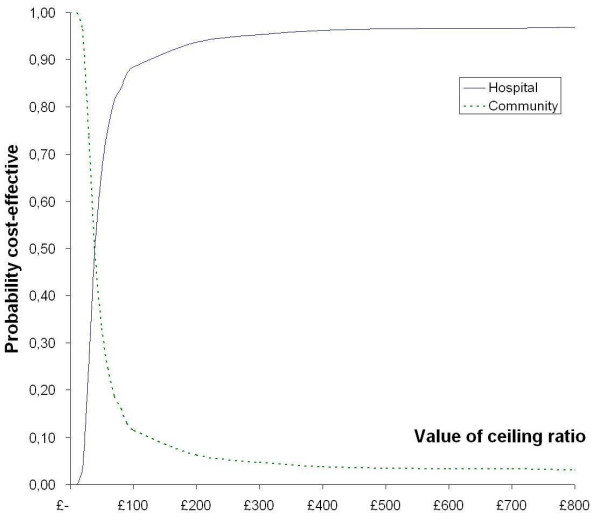
**Cost-effectiveness acceptability curve**. This shows the probability that one setting is more cost-effective than another for a given ceiling value and for the assumption that prevalence in hospital sites is higher than in community sites. QCM = quality weighed detected child months.

Higher prevalence in hospital resulted in higher costs (figure 3) and higher amount of QCM. Figure 4 shows the cost-effectiveness acceptability curve for the assumption that prevalence in hospital was higher than in community sites (0.002 in H versus 0.001 in C). If decision makers were willing to pay at least £ 500 per QCM gained, the probability of hospital being more cost-effective under this assumption would be 95%. If the willingness to pay was below £ 30 per QCM, community sites were more cost-effective with a probability of 95%. For the assumption that prevalence in community sites was higher than in hospital sites, community sites were more cost-effective for any ceiling ratio. Any difference in sensitivity predicted differences in costs. Higher sensitivity in any site resulted in higher costs. If decision makers were willing to pay at least £ 300 per QCM gained, the probability of hospital being more cost-effective under the assumption of higher sensitivity in hospital would be 95%. If willingness to pay were below £ 150 per QCM, community sites would be more cost-effective with a probability of 95%. For the assumption that screen sensitivity in community sites was higher than in hospital sites, community sites were more cost-effective for any ceiling ratio. Low coverage resulted in low costs. With coverage in hospital being higher than in community, community settings would be more cost-effective with a probability of 95% if willingness to pay were below £ 350 per QCM. With coverage in hospital being lower, hospital settings would be more cost-effective with a probability of 95% if willingness to pay were below £ 200. Differences in screen specificity between hospital and community sites resulted in cost differences but not in effectiveness differences. Higher specificity resulted in lower costs. Any differences in costs per screening procedure resulted in output cost differences. With all other parameters held constant in both settings, variance in input costs completely predicted variance in output costs.

## Discussion

We applied a decision-analytic Markov model to empiric data of first stage implementation areas of NHSP in England to evaluate cost and effectiveness of different settings for newborn hearing screening. Base case assumptions with constant test parameters but cost difference between hospital and community settings yielded a cost difference of £25 per quality weighted detected child month (QCM): To detect one hearing impaired child one month earlier produced costs of £268 in hospital settings and of £243 in community settings. This cost difference, however, was not statistically significant. Probabilistic multivariate Monte Carlo simulation revealed that in nearly half of 1000 simulated trials community settings would yield higher costs than hospital settings. The cost-effectiveness of the two newborn screening models – hospital-based and community-based – did not differ significantly, assuming comparable screen performance for the two newborn screening models. Projected magnitude of costs per detected child was comparable to the costs found by other UNHS models [23], proving the model to give results of external validity. As this is the first model to report costs per quality weighted child month, these results can not be directly compared to other findings.

Up to now preliminary data were too sparse to detect any differences of important input parameters – like screen performance and program costs – between settings. Extremes analyses showed that any statistically significant difference in prevalence, sensitivity, specificity and costs would result in significant differences in cost-effectiveness between settings. Any further evaluation of cost-effectiveness between different programme alternatives should evaluate in the first place if there is substantial difference in terms of these parameters.

Our study has several limitations. Even though QCM was weighted, it is a surrogate parameter for the actual burden of disease for the child. To date, however, there is no study yielding empirical data on a more general effectiveness measure such as quality adjusted life years. There are drawbacks of this study concerning uncertainty on model parameters. There is still only limited evidence for further benefits of early diagnosis and intervention. Weighting assumptions in the presented model are therefore only estimates of the potential impact of late diagnosis and the actual child's burden of disease. To date there is no further studies should be conducted on this issue. Other parameter uncertainties should be ruled out as soon as long term data from the NHSP implementation are available.

Probabilistic analysis of the incremental cost-effectiveness ratio can be used to give ceiling values. Policy makers can then decide on a fixed incremental effectiveness they would like to obtain by introducing a screening program and the model will show how probable this outcome will be under the assumption of parameter uncertainty.

As a part of ongoing and future research the NHSP Evaluation aims to identify factors predicting high cost-effectiveness for either model and to compare maternal satisfaction/anxiety in hospital-based and community-based setting.

## Conclusion

The value of this modelling exercise lies in the provision of information to decision makers by a quantitative projection of available data and the explicit and transparent statements about assumptions and the degree of uncertainty. This has been achieved at an early stage of implementation. The evaluation of the NHS Newborn Hearing Screening Programme will serve as a valuable tool and example to justify and improve large scale screening programmes.

## Competing interests

The author(s) declare that they have no competing interests.

## Authors' contributions

EG carried out the statistical analyses and drafted the manuscript. KU designed and coordinated the study and participated in the writing process. FH, RST and JW participated in the statistical analyses and in the writing process. LD participated in the design of the study. JB conceived of the study and participated in its design and coordination. All authors read and approved the final manuscript.

## Pre-publication history

The pre-publication history for this paper can be accessed here:



## Figures and Tables

**Table 1:** Data input for the model

Estimated parameter	Setting	Baseline estimate	Range for sensitivity analysis	Extremes	Source
Prevalence of newborn hearing impairment %	H C	0.15	0.09–0.3	0.01–0.2 0.01–0.2	Literature [3][4][14][15][16]
Sensitivity of screening %	H C	96	96–100	70–99 70–99	Literature [10]
Specificity of screening %	H C	99	99	70–99	data from sites, calculated
Coverage of screening %	H C	97	97	50–99	data from sites
Follow-up after screening %	H C	95	95		Authors' estimate
Healthy children under suspicion of hearing impairment %	H C	0.1	0.1		Authors' estimate
Discounting factor Costs %	H C	6 per year 6 per year			
Effects %	H C	1.5 per year 1.5 per year			
Probability of "natural" discovery without systematic screening	H C	Distribution, smoothed Weibull curve	Median age at diagnosis 18 months		Empirical data [18]
Costs of screening per child	H C	£ 35.58 (32–40)	£ 31.99 (29–35)	£ 28–59 £ 27–43	Data from sites
Costs of audiological follow-up of referrals	H C	£ 160	£ 160		Estimate from sites

H = hospital C = community

**Table 2:** Annual birth rates of participating areas

Area	Birth rate per 1,000 inhabitants
Avon	11.7
Barnsley	11.7
Bradford	14.5
Bucks	12.7
Calderdale & Huddersfield	13.1
Camden & Islington	14.1
Dewsbury	13.1
East London & City	17.8
East Sussex	12.6
Manchester	13.5
North Cheshire	12.3
North Derbyshire	10.8
North Staffordshire	11.0
Northumberland	11.6
Nottingham	11.5
Redbridge & Waltham Forest	14.8
Oxford	12.6
Sheffield	11.4
Shropshire	11.8
Southampton	11.2
Stockport	11.2
Wiltshire (Bath)	10.6
Wiltshire (Swindon)	12.6

**Table 3:** Model results base case assumption (discounted) for a hypothetical cohort of 100,000 children

outcome	Alternative settings hospital	community	incremental
	Base case	Base case	

effects			
QCM at 6 months	794	794	
QCM at 12 months	1536	1536	
QCM at 120 months	13751	13751	
TP at 6 months	134	134	
TP at 120 months	150	150	
FP after screening and additional diagnostic	12	12	
costs			
Costs per 100,000 at 120 months	£ 3.690.022	£ 3.343.572	£ 346.450
Cost per detected child	£ 25.813	£ 23.390	£ 2423
Cost per QCM	£ 268	£ 243	£ 25

QCM = quality weighed detected child months

TP = true positives

FP = false positives

**Table 4:** One-way sensitivity analyses

Item	Hospital site Cost per QCM	Community site Cost per QCM	Incremental
Base case	£ 268	£ 243	25
Prevalence (%)			
low (0.09)	£ 437	£ 395	42
high (0.3)	£ 142	£ 129	13
Sensitivity (%)			
low (0.96)	£ 268	£ 243	25
high (100)	£ 263	£ 239	24
Costs (£)			
low (H 32, C 29)	£ 243	£ 222	21
high (H 40, C 35)	£ 299	£ 264	35

QCM = quality weighed detected child month, detected child months weighted by a utility value indicating the prognosis of further speech development

H = Hospital

C = Community
